# Ankle tibiotalocalcaneal nailing in elderly ankle fractures as an alternative to open reduction internal fixation: technique and literature review

**DOI:** 10.1097/OI9.0000000000000183

**Published:** 2022-07-18

**Authors:** Apostolos Dimitroulias

**Affiliations:** a Division of Orthopaedic Surgery, NYC Health+Hospitals/Jacobi; b Department of Orthopaedic Surgery, The Albert Einstein College of Medicine, Montefiore Medical Center, Bronx, NY.

**Keywords:** ankle fracture, elderly, geriatric, hindfoot nail, tibiotalocalcaneal nail

## Abstract

The use of tibiotalocalcaneal nails for unstable ankle fractures in low demand elderly patients has been introduced as an alternative to open reduction internal fixation to allow early weight-bearing and to decrease soft tissue complications and mechanical failures. This paper describes the technique of hindfoot nailing and reviews the current literature. Overall, it is a minimally invasive and expeditious procedure that provides stable fixation to withstand immediate ambulation of the frail elderly patient. Future high-quality randomized controlled trials will determine if complications and outcomes compare favorably to open reduction and internal fixation.

## 1 Introduction

Elderly patients are often not able to mobilize with non-weight bearing or partial-weight bearing restrictions on a lower extremity due to poor health, decreased stamina, and problems with motor coordination and balance. Therefore, it would be beneficial for elderly patients with ankle fractures to initiate early weight bearing and rehabilitation. Recent studies support early weight bearing after open reduction and internal fixation of ankle fractures; however, these studies have been in patients younger than 65.^[1–4]^ There is an increasing body of literature that for elderly frail patients with unstable ankle fractures and comorbid conditions such as osteoporosis, chronic kidney disease, and complicated diabetes, the use of a tibiotalocalcaneal (TTC) nail is an expedited procedure that allows early weight bearing with decreased complications and satisfactory outcomes.^[5–15]^ Unlike arthrodesis done for arthritis, the goal of this technique is not to formally fuse the tibiotalar (TT) and subtalar (ST) joints, but to provide stability to the ankle for immediate unrestricted weight bearing as an “internal splint.” Therefore, preparation of the joints is not usually required. In these low-demand patients, sacrificing the motion to the TT and ST joints has not been shown to be disabling.

## 2 Technique description

**Figure 1:**
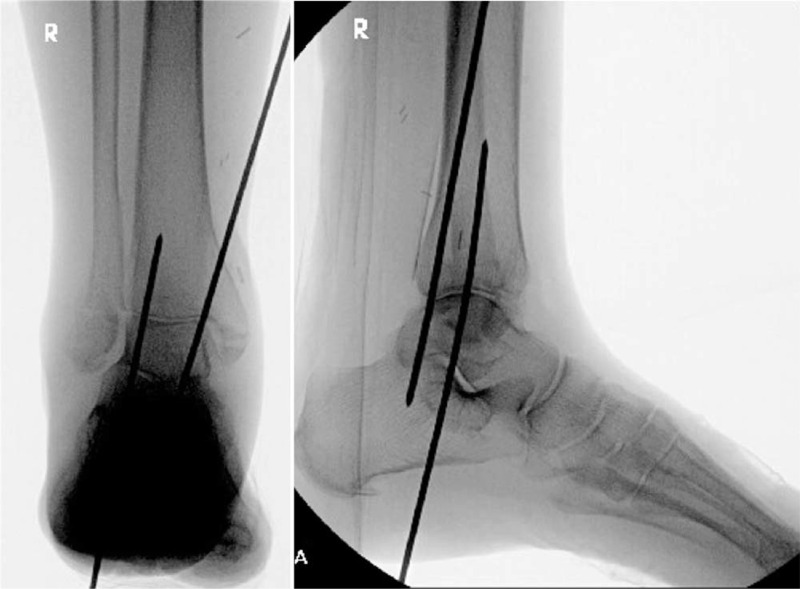
The ankle fracture is reduced, and with the foot plantigrade at neutral dorsiflexion, 5 degrees valgus and 5 degrees external rotation, the wires are advanced across the tibiotalar joint away from the predicted nail path.

The patient is placed supine. The ideal position for ankle fusion is with the foot in neutral dorsiflexion/plantarflexion, 5 degrees of hindfoot valgus and equal or slightly more external rotation of the foot compared with the opposite side. The anterior cortex of the distal tibia should be in line with the anterior edge of the talar dome. With closed maneuvers, the talus is reduced under the tibia and when the ideal position of the foot in relation to the tibia has been achieved, Kirschner wires are driven from the calcaneus to talus and tibia, away from the expected nail path (Fig. 1).

**Figure 2:**
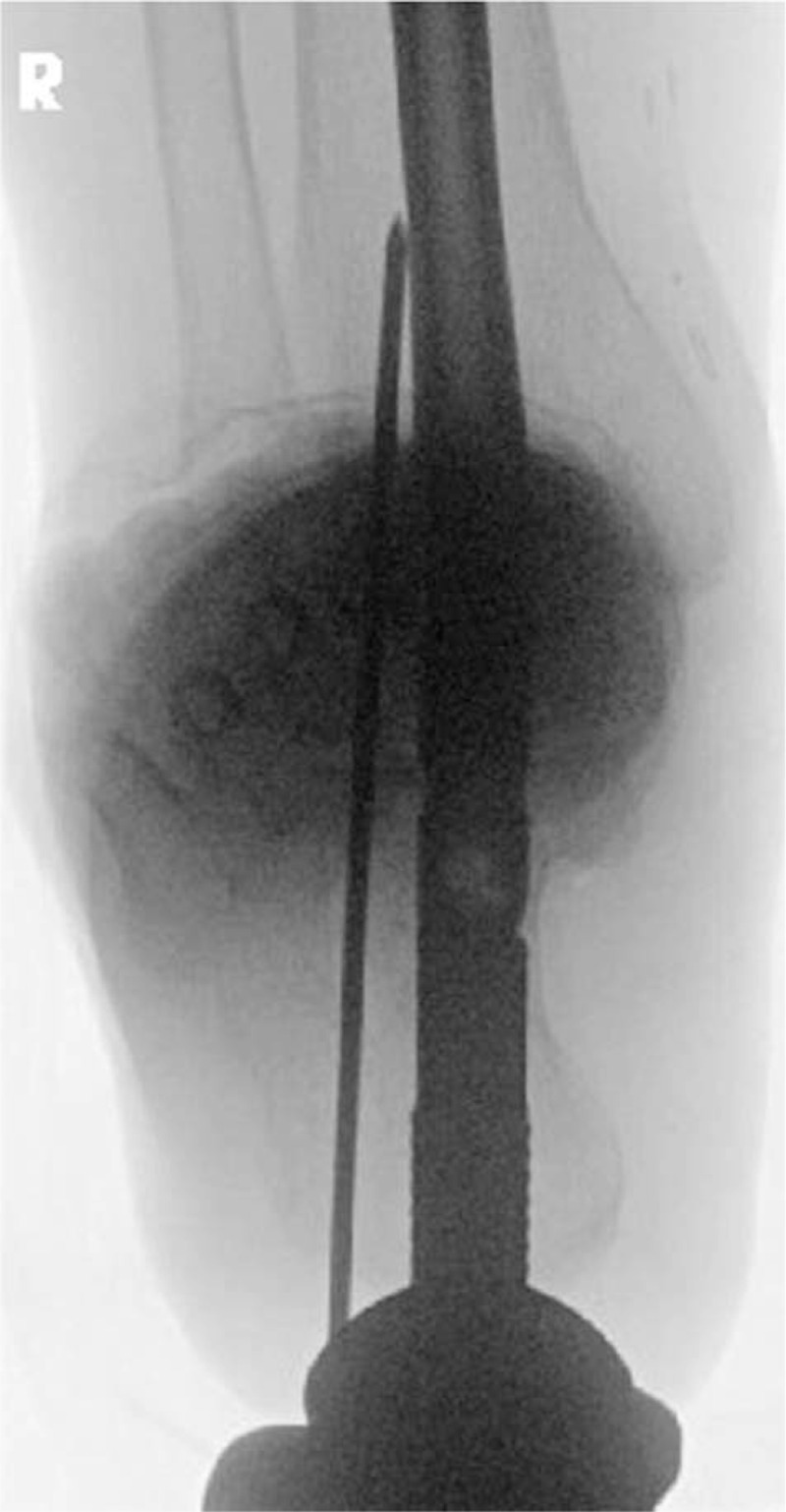
Hindfoot alignment view demonstrating that the nail entry site is not violating the medial cortex of the calcaneus body near the sustentacular area.

**Figure 3:**
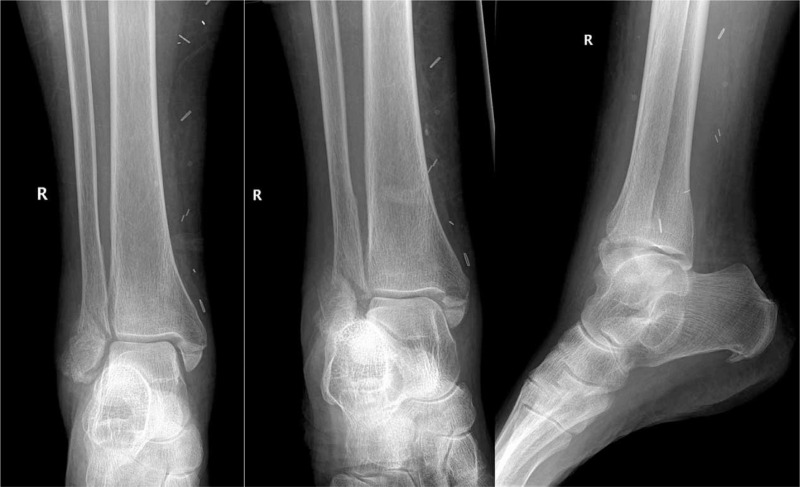
A 71-year-old female sustained a ground level fall. Past medical history included diabetes, essential thrombocytosis, cerebrovascular accident, peripheral arterial disease, coronary artery disease, heart failure, asthma, and fibromyalgia. She had low functional status ambulating less than 2 blocks with an unsteady gait. She sustained an AO/OTA 44B2.3 injury (transsyndesmotic multifragmented fibula fracture and medial malleolus fracture). Due to the peripheral arterial disease the soft tissue envelope was not amenable for ORIF. Also the fibula fracture was comminuted and not amenable for intramedullary fixation.

**Figure 4:**
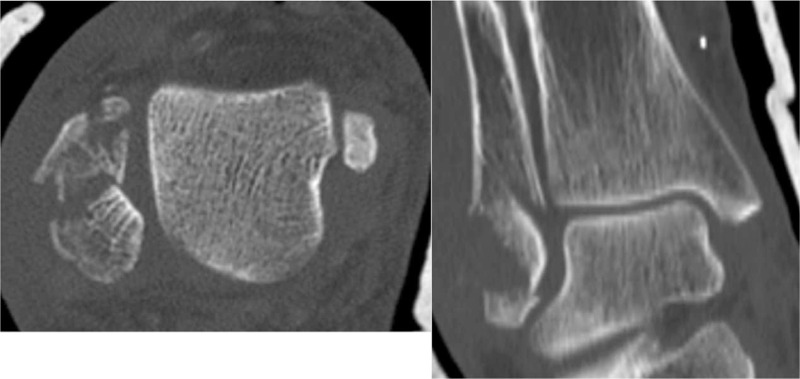
CT scan revealing the multifragmented nature of distal fibula.

**Figure 5:**
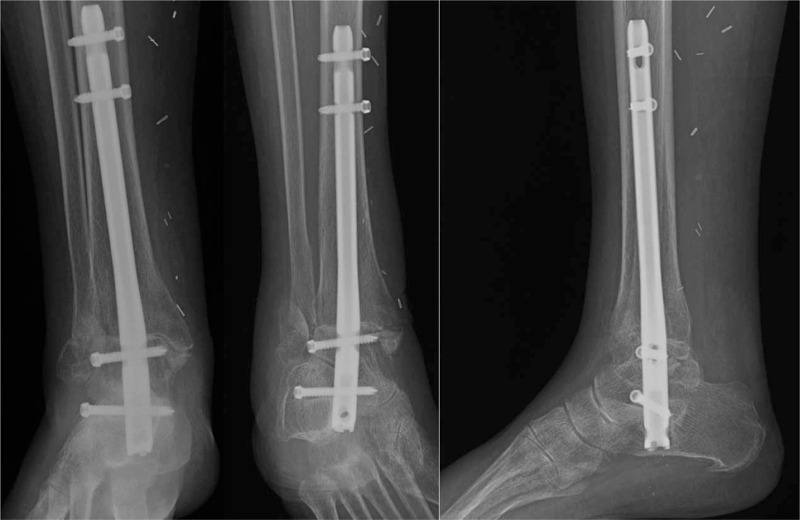
Patient underwent fixation with a tibiotalocalcaneal nail and allowed early weight-bearing. At 3.5months postoperatively, radiographs reveal stable hardware and healing lateral and medial malleolus fractures.

The body of the calcaneus is not in line with the tibia on the coronal plane. Modern nails have a lateral (valgus) curve that allows the entry point to be lateralized in line with the midcalcaneal axis^[16]^ or slightly lateral to the midline of the heel.^[17]^ This provides better bony anchorage in the calcaneus, decreases the risk of damage of the lateral plantar neurovascular bundle, and assists in achieving the 5 degrees valgus alignment of the hindfoot. If a straight nail were used, it would require a more medial starting point close to the sustentaculum area. On the sagittal plane, the entry point is in line with the tibia IM canal, as this is marked on the lateral fluoroscopic image. To ensure that the guide wire is not too medial in the calcaneus in the coronal plane, the hindfoot alignment view^[18]^ can be used. Intra-operatively, this view can be obtained by tilting the C-arm 20 degrees caudal-cranially with the foot in neutral position with toes pointing up (Fig. 2). The guide wire is placed on the plantar aspect of the calcaneus body using all 3 views (anteroposterior, lateral, and hindfoot alignment) and then advanced through the calcaneus to the center of the talar dome and tibial plafond using AP and lateral ankle imaging. A 2-3 cm incision is made in line with the guide wire. The nail pathway is gradually opened with reamers up to a diameter 1 mm larger than the desired nail diameter. To avoid stress concentration and fracture around the tip of the retrograde nail, short nails (150 mm) are not recommended.^[19]^ Longer nails (300 mm+) that end at the isthmus of the tibia or more proximal do not allow for toggling in the coronal plane and provide greater stability to the nail.^[19,20]^ The depth of insertion is monitored on the lateral projection to ensure that the distal part of the dynamic hole is in the talus and that the distal tip of the nail is not prominent. Proximal locking is done first. Then, a locking screw through the dynamic position is inserted in the talus and following that the nail-mounted internal compression screw is tightened against the talar screw. Although this step is optional, we prefer the use of second-generation nails that allow for internal compression as sustained compression of the tibiotalar surfaces would increase the stability of the construct.^[21]^ Further locking screws are inserted depending on the nail design and surgeon's preference. A soft dressing is applied and the patient is allowed to start unrestricted weight bearing (Figs. 3–5).

## 3 Literature review

In all published studies of elderly ankle fractures treated with a TTC nail, no formal preparation of TT and/or ST joint was performed.^[5–15]^ It is believed that the absence of motion, cartilage compression, and the reaming process will stimulate the fusion across the subtalar and tibiotalar joints. For those patients in whom fusion does not occur, hardware failure is not seen very often due to the sedentary activity level, with nail breakage rates reported up to 6.4%.^[12]^ Moreover, in frail elderly patients with multiple comorbidities and a compromised soft tissue envelope, the risks of a lengthier procedure with extra soft tissue dissection to formally debride the TT and ST joints outweigh the benefits. In younger more active patients, formal fusion is required to prevent hardware failure. Other hardware-related complications include screw loosening or breakage up to 9.6%.^[22]^ Periprosthetic fracture at the tip of the nail is a unique complication that can occur after hindfoot nailing, and has been reported in up to 9.6%.^[12]^ This is attributed to the bending forces at the distal tibia area due to a stiffer foot and ankle. For this reason, it is recommended to use longer nails to avoid strain concentration in this area.^[19,20]^ The risk of deep infection ranges from 0% to 11% with the upper range seen in a study where all the patients had complicated diabetes.^[9]^ The rate of symptomatic fracture nonunion in published studies varies from 0% to 12%.^[5,9,10,12,15]^

Return to preinjury level of mobility was observed in 75% to 100% of the patients in different studies.^[5,6,8–12]^ This high percentage of patients returning to function may be due to earlier full weight-bearing but also due to the low level of preinjury activity.

In comparison, open reduction and internal fixation of ankle fractures in the elderly has high rates of complications, up to 20%.^[23]^ In the presence of systemic comorbidities such as diabetes, peripheral vascular disease, neuropathy, smoking, obesity, osteoporosis or with severe soft tissue injury, comminution and bone loss, the complication rate increases to over 40%.^[24,25]^

Only 1 randomized trial to date has compared TTC versus ORIF in elderly ankle fractures. It found a significant difference in postoperative complications favoring TTC (8.1 vs 33.3%), a shorter hospital stay, and similar outcomes regarding return to preinjurystate and postoperative OMASscore.^[10]^ This studyhad some discrepancies in data presentation (conflicting number of patients) and there is no description of the randomization method. Currently, there is an ongoing multicenter prospective randomized controlled trial comparing complications and outcomes of elderly ankle fractures treated with TTC versus ORIF.^[26]^

In conclusion, the use of a TTC nail for the low functioning elderly with systemic comorbidities provides immediate stable fixation, allows early full weight bearing, is minimally invasive, can be done expeditiously, and has an acceptable complication profile. Future higher quality studies will provide the orthopaedic community with better insight regarding its use.

## References

[1] Dehghan N McKee MD Jenkinson RJ Early weightbearing and range of motion versus non-weightbearing and immobilization after open reduction and internal fixation of unstable ankle fractures: a randomized controlled trial. *J Orthop Trauma* 2016; 30:345–352. 27045369 10.1097/BOT.0000000000000572

[2] Park JY Kim BS Kim YM Early weightbearing versus nonweightbearing after operative treatment of an ankle fracture: a multicenter, noninferiority, randomized controlled trial. *Am J Sports Med* 2021; 3635465211026960. 10.1177/03635465211026960 34251882

[3] Sernandez H Riehl J Fogel J. Do early weight-bearing and range of motion affect outcomes in operatively treated ankle fractures: a systematic review and meta-analysis. *J Orthop Trauma* 2021; 35:408–413. 33512860 10.1097/BOT.0000000000002046

[4] Smeeing DPJ Houwert RM Briet JP Weight-bearing or non-weight-bearing after surgical treatment of ankle fractures: a multicenter randomized controlled trial. *Eur J Trauma Emerg Surg* 2020; 46:121–130. 30251154 10.1007/s00068-018-1016-6 PMC7026225

[5] Al-Nammari SS Dawson-Bowling S Amin A Fragility fractures of the ankle in the frail elderly patient: treatment with a long calcaneotalotibial nail. *Bone Joint J* 2014; 96-B:817–822. 24891584 10.1302/0301-620X.96B6.32721

[6] Amirfeyz R Bacon A Ling J Fixation of ankle fragility fractures by tibiotalocalcaneal nail. *Arch Orthop Trauma Surg* 2008; 128:423–428. 18270721 10.1007/s00402-008-0584-z

[7] Armstrong L Jackson J Riddick A. Tibiotalocalcaneal nail fixation and soft tissue coverage of Gustilo-Anderson grade 3B open unstable ankle fractures in a frail population; a case series in a major trauma centre. *Foot Ankle Surg* 2018; 24:347–352. 29409236 10.1016/j.fas.2017.03.015

[8] Baker G Mayne AIW Andrews C. Fixation of unstable ankle fractures using a long hindfoot nail. *Injury* 2018; 49:2083–2086. 30185373 10.1016/j.injury.2018.07.028

[9] Ebaugh MP Umbel B Goss D Outcomes of primary tibiotalocalcaneal nailing for complicated diabetic ankle fractures. *Foot Ankle Int* 2019; 40:1382–1387. 31423816 10.1177/1071100719869639

[10] Georgiannos D Lampridis V Bisbinas I. Fragility fractures of the ankle in the elderly: open reduction and internal fixation versus tibio-talo-calcaneal nailing: short-term results of a prospective randomized-controlled study. *Injury* 2017; 48:519–524. 27908492 10.1016/j.injury.2016.11.017

[11] Herrera-Perez M Martin-Velez P Rendon-Diaz D Acute retrograde tibiotalocalcaneal nailing in osteoporotic periarticular ankle fractures. *J Foot Ankle* 2020; 14:117–122.

[12] Jonas SC Young AF Curwen CH Functional outcome following tibio-talar-calcaneal nailing for unstable osteoporotic ankle fractures. *Injury* 2013; 44:994–997. 23237604 10.1016/j.injury.2012.11.008

[13] Lemon M Somayaji HS Khaleel A Fragility fractures of the ankle: stabilisation with an expandable calcaneotalotibial nail. *J Bone Joint Surg Br* 2005; 87:809–813. 15911664 10.1302/0301-620X.87B6.16146

[14] Persigant M Colin F Noailles T Functional assessment of transplantar nailing for ankle fracture in the elderly: 48 weeks’ prospective follow-up of 14 patients. *OrthopTraumatolSurg Res* 2018; 104:507–510. 10.1016/j.otsr.2018.03.008 29654935

[15] Taylor BC Hansen DC Harrison R Primary retrograde tibiotalocalcaneal nailing for fragility ankle fractures. *Iowa Orthop J* 2016; 36:75–78. 27528840 PMC4910785

[16] Lee BH Fang C Kunnasegaran R Tibiotalocalcaneal arthrodesis with the hindfoot arthrodesis nail: a prospective consecutive series from a single institution. *J Foot Ankle Surg* 2018; 57:23–30. 29129314 10.1053/j.jfas.2017.05.041

[17] Moorjani N Buckingham R Winson I. Optimal insertion site for intramedullary nails during combined ankle and subtalar arthrodesis. *Foot Ankle Surg* 1998; 4:21–26.

[18] Saltzman CL el-Khoury GY. The hindfoot alignment view. *Foot Ankle Int* 1995; 16:572–576. 8563927 10.1177/107110079501600911

[19] Noonan T Pinzur M Paxinos O Tibiotalocalcaneal arthrodesis with a retrograde intramedullary nail: a biomechanical analysis of the effect of nail length. *Foot Ankle Int* 2005; 26:304–308. 15829214 10.1177/107110070502600406

[20] Wukich DK Mallory BR Suder NC Tibiotalocalcaneal arthrodesis using retrograde intramedullary nail fixation: comparison of patients with and without diabetes mellitus. *J Foot Ankle Surg* 2015; 54:876–882. 26015305 10.1053/j.jfas.2015.02.019 PMC5664154

[21] Berson L McGarvey WC Clanton TO. Evaluation of compression in intramedullary hindfoot arthrodesis. *Foot Ankle Int* 2002; 23:992–995. 12449401 10.1177/107110070202301103

[22] Taylor J Lucas DE Riley A Tibiotalocalcaneal arthrodesis nails: a comparison of nails with and without internal compression. *Foot Ankle Int* 2016; 37:294–299. 26472084 10.1177/1071100715611891

[23] Zaghloul A Haddad B Barksfield R Early complications of surgery in operative treatment of ankle fractures in those over 60: a review of 186 cases. *Injury* 2014; 45:780–783. 24388418 10.1016/j.injury.2013.11.008

[24] McCormack RG Leith JM. Ankle fractures in diabetics. Complications of surgical management. *J Bone Joint Surg Br* 1998; 80:689–692. 9699839 10.1302/0301-620x.80b4.8648

[25] Pearce O Al-Hourani K Kelly M. Ankle fractures in the elderly: current concepts. *Injury* 2020; 51:2740–2747. 33153712 10.1016/j.injury.2020.10.093

[26] Tuckett P Hope M Tetsworth K Van De Pol J McDougall C. Transarticular tibiotalocalcaneal nailing versus open reduction and internal fixation for treatment of the elderly ankle fracture: protocol for a multicentre, prospective, randomised controlled trial. *BMJ Open* 2019; 9:e026360. 10.1136/bmjopen-2018-026360 PMC6347874 30670529

